# Hyaluronic Acid-Modified and TPCA-1-Loaded Gold Nanocages Alleviate Inflammation

**DOI:** 10.3390/pharmaceutics11030143

**Published:** 2019-03-25

**Authors:** Jingnan Zhao

**Affiliations:** Biotechnology G2018, School of International Education, Henan University of Technology, No. 100 Lianhua Street, Zhengzhou 450001, China; 201820040221@stu.haut.edu.cn; Tel.: +86-187-0399-6403

**Keywords:** gold nanocage, inflammation, TPCA-1, hyaluronic acid, TNF-α, IL-6, rheumatoid arthritis

## Abstract

Gold nanocages (AuNCs) are biocompatible and porous nanogold particles that have been widely used in biomedical fields. In this study, hyaluronic acid (HA) and peptide- modified gold nanocages (HA-AuNCs/T/P) loaded with 2-[(aminocarbonyl)amino]-5-(4-fluorophenyl)-3-thiophenecarboxamide (TPCA-1) were prepared to investigate their potential for combating inflammation. TPCA-1 was released from AuNCs, intracellularly when HA was hydrolyzed by hyaluronidase. HA-AuNCs/T/P show a much higher intracellular uptake than AuNCs/T/P, and exhibit a much higher efficacy on the suppression of tumor necrosis factor alpha (TNF-α) and interleukin 6 (IL-6) than free TPCA-1, suggesting great improvement to the anti-inflammatory efficacy of TPCA-1 through the application of AuNCs. HA-AuNCs/T/P can also reduce the production of reactive oxygen species in inflammatory cells. This study suggests that HA-AuNCs/T/P may be potential agents for anti-inflammatory treatment, and are worthy of further investigation.

## 1. Introduction

Rheumatoid arthritis (RA) is an autoimmune disease that may lead to the damage of cartilage and bone, loss of mobility, organ failure, and death. Although the pathogenesis of RA is not totally understood, RA is characterized by multiple inflamed joints. Past experience indicates that successful suppression of inflammation halts the progression of RA. Proinflammatory cytokines, such as tumor necrosis factor alpha (TNF-α), interleukin 6 (IL-6), and vascular endothelial growth factor (VEGF), play an important role in aggravating and sustaining joint inflammation [1]. RA pathogenesis also includes the dysregulation of signaling pathways in inflamed cells. Nuclear factor κB (NF-κB) is one of the most important signaling pathways that are activated in inflammation [2,3]. 2-[(aminocarbonyl)amino]-5-(4-fluorophenyl)-3-thiophenecarboxamide (TPCA-1), acts as an inhibitor of IκB kinases (IKK), which are involved in the activation of NF-κB signaling pathways. TPCA-1 can inhibit NF-κB signaling pathways and, subsequently, suppress the transcription of TNF-α, IL-6, and other proinflammatory cytokines [4,5].

Activated macrophages play a vital role in RA pathophysiology. CD44 is an overexpressed receptor on activated macrophages that is a potential target site for RA treatment. Hyaluronic acid (HA), a natural polysaccharide, exhibits desirable biocompatibility and biodegradability, and has been extensively applied in novel drug-delivery systems. Most importantly, HA is a targeting moiety for CD44 receptors and has been successfully applied in many studies [6,7,8]. Hyaluronidase (Hyal), which specifically degrades hyaluronan, is a lysosomal enzyme [7]. Hyal levels in the RA synovium are significantly greater than in healthy synovium.

Gold nanocages (AuNCs) are biocompatible and porous nanogolds that have been widely used in the biomedical field for imaging [9] and drug delivery [10]. AuNCs have been used as drug carriers for anticancer treatments and have shown significant inhibition effects on tumor growth in vitro and in vivo [7,11]. However, there have been no reports regarding the anti-inflammatory application of AuNCs. Gold compounds have been used for the treatment of inflammatory diseases, such as RA, for several decades [12]. Gold nanoparticles have also been explored for their use in the treatment of RA, because they reduce the production of reactive oxygen species (ROS) in response to the receptor activation of NF-κB ligand in macrophages [13]. Several studies have demonstrated the dual functionality of nanogold as both drug carriers and therapeutics [3,13,14]. Therefore, AuNCs may have unexplored advantages for anti-inflammatory applications.

In this study, AuNCs were used to load TPCA-1 and the drug-loaded AuNCs were then modified with a positively charged peptide and HA. Positively charged peptides can combine with negatively charged AuNCs and subsequently increase the loading capacity of TPCA-1 to AuNCs. HA acts as a targeting moiety and an enzyme-responsive gatekeeper for TPCA-1 release. When HA-AuNCs/T/P meet an inflammatory microenvironment or lysosome, contact is made with Hyal, resulting in the degradation of HA and the release of TPCA-1. 

## 2. Materials and Methods

### 2.1. Reagents and Cell Lines

2-[(aminocarbonyl)amino]-5-(4-fluorophenyl)-3-thiophenecarboxamide (TPCA-1; CAS. no. 507475-17-4) was purchased from Abmole Bioscience Inc. (Houston, TX, USA). Alfa Chemicals (Pune, India) provided sodium borohydride, chloroauric acid trihydrate (HAuCl_4_·3H_2_O), silver nitrate (AgNO_3_), and sodium citrate. Synthetic peptides, such as MCKYFIKIVSKSAKKPVGLIGC and MCKYFIKIVSKSAKK(FITC)PVGLIGC (FITC-labeled peptide), were obtained from DGpeptides Co., Ltd (Hangzhou, China). The purity of all the synthetic peptides was >99.0%. RAW 264.7 cell line was provided by ATCC (Manassas, VA, USA). Solarbio (Beijing, China) provided 4′,6-diamidino-2-phenylindole (DAPI), RPMI 1640 cell culture medium, ROS assay kit, and mouse TNF-α ELISA Kit. The mouse IL-6 ELISA Kit was purchased from Liankebio (Hangzhou, China). Fetal bovine serum (FBS) for cell culture was obtained from Biological Industries Ltd (Beit Haemek, Israel). 

### 2.2. Synthesis of AuNCs

AuNCs were synthesized according to a previously reported method, with some modifications [7,15]. Briefly, a mixture of 4 mL of 1% (*w*/*v*) sodium citrate solution and 15 mL of deionized water (dH_2_O) was added to a round-bottom flask and heated to 70 °C for 15 min. Subsequently, 340 μL of 1% (*w*/*v*) AgNO_3_ was introduced into the mixture, followed by the immediate addition of 400 μL of 0.1% (*w*/*v*) freshly prepared sodium borohydride solution. Under vigorous stirring, the reaction solution was kept at 70 °C for 1 h and then cooled to room temperature. Silver nanoparticles (AgNPs), with an average particle size of 4.0 nm, were obtained.

The stepwise seeding growth method was utilized to obtain AgNPs seeds of AuNCs synthesis. A mixture of 4.0 mL of 1% (*w*/*v*) sodium citrate solution and 15 mL of dH_2_O was heated to boiling for 15 min. Subsequently, 2 mL of 4.0 nm AgNPs solution was added into the mixture under vigorous stirring for 1 h, while maintaining reflux, after which 340 μL of 1% (*w*/*v*) AgNO_3_ solution was also added. Following this, 400 μL of 1% sodium citrate solution was added to the reaction solution with 340 μL of 1% AgNO_3_ solution under vigorous stirring for another 1 h, while maintaining reflux. The same operation was repeated again. Finally, the reaction solution was cooled to room temperature in order to obtain AgNP seeds. 

AgNP seeds were subsequently used to synthesize AuNCs. First, 10 mL of 1.0 mg/mL polyvinylpyrrolidone (PVP) were added to a round-bottom flask and kept at 90 °C for 1 h, under vigorous stirring. After that, 1.0 mL of AgNP seeds solution was introduced to the mixture, followed by the addition of 4.0 mL of HAuCl_4_ (0.0711 mg/mL) at a rate of 0.75 mL/min, until the color (blue) of the reaction was stable. The solution was centrifuged and washed with a saturated NaCl solution in order to remove AgCl, and with water several times to remove PVP and NaCl when the solution was cooled to room temperature. After centrifugation following the last wash, the precipitate (AuNCs) was suspended with 5.0 mL of dH_2_O and the concentration of AuNCs was determined using inductively coupled plasma atomic emission spectroscopy (iCAP 6500 DUO, Thermo Fisher Scientific, Shanghai, China). The concentration of AuNCs was approximately 12 μg/mL.

### 2.3. TPCA-1 Loading

TPCA-1 was first loaded inside the AuNCs as follows: 1.5 mL of AuNCs solution (12 μg/mL) was centrifuged at 13,500 rpm for 15 min at 4 °C, and the supernatant was removed. The sediment (AuNCs) was suspended in 1.0 mL of peptide solution (50 μg/mL) and incubated for 2 h under stirring (70 rpm) at 37 °C, followed by centrifuging and washing with dH_2_O to remove any residual free peptide. After that, the sediment was suspended in 0.5 mL of dH_2_O, followed by the addition 0.2 mL of aqueous DMSO (DMSO/dH_2_O = 2:3 (*v*/*v*)) solution of TPCA-1 (50 μg/mL), and the mixture was vortexed for 3 min. Then, 0.2 mL of HA (400 μg/mL) was added to the mixture for 2 h under stirring (70 rpm) at 37 °C, followed by centrifuging and washing with dH_2_O to remove any residual free TPCA-1 and HA. The obtained pellet was suspended and diluted, based on the requirements of the experiment. All of the washing solutions were collected, and the loading of TPCA-1 was calculated from the difference in the concentrations between the initial and the resulting free TPCA-1.

### 2.4. Measurements and Characterizations

Particle size, polydispersity (PDI), and zeta potential of AuNCs, AuNCs/T/P, and HA-AuNCs/T/P in dH_2_O were measured using a Zetasizer Nano ZS (Malvern, UK). A total of 20 μL of AuNCs or HA–AuNCs/T/P was pipetted onto a carbon-coated copper grid (Beijing Xinxing Braim Technology Co., Ltd, Beijing, China). The excess sample solution was removed, and after the sample was dried, the images of AuNCs and HA-AuNCs/T/P were recorded using a transmission electron microscope (TEM, TecnaiG20, FEI, USA) operating at 200 KV. To investigate the action of Hyal for the release of TPCA-1, the HA-AuNCs/T/P were dispersed in phosphate buffer solutions (pH 7.4) with Hyal (150 U/mL) under stirring at 37 °C for 4 h. Subsequently, the released samples were centrifuged at 13,500 rpm for 15 min at 4 °C. The concentration of released TPCA-1 was determined using a UV-2700 spectrometer (Shimadzu, Japan) at a wavelength of 310 nm, based on the standard curve. UV–Vis absorption spectrum of AuNCs, AuNCs/T/P, and HA-AuNCs/T/P were also recorded using a UV-2700 spectrometer (Shimadzu, Japan).

### 2.5. Cellular Uptake of HA-AuNCs/T/P

The FITC-labeled peptides were used to investigate the cellular uptake of HA-AuNCs/T/P using a Nikon A1 confocal microscope (Nikon, Japan) to observe RAW 264.7 cells. Briefly, AuNCs/T/P (10 μg/mL) and HA-AuNCs/T/P (10 μg/mL) were added to RAW 264.7 cells (cell density 7.5 × 10^4^ cells/well) in 24-well plates and incubated for 4 h. Afterwards, the medium in each well was removed and cells were washed three times with PBS. Five hundred microliters of 4% paraformaldehyde was added to each well and the cells were incubated for 10 min in the dark. Subsequently, cells were washed three times with PBS, and 500 μL of DAPI solution (10 μg/mL) was used to stain the cells for 5 min. Afterwards, the cells were washed three times again using PBS, and then observed under the confocal microscope. 

### 2.6. In Vitro Anti-Inflammatory Efficacy in RAW 264.7 Cells

RAW 264.7 cells were plated at 1.5 × 10^4^ cells/well in 96-well tissue culture plates and incubated for 24 h at 37 °C. Adhered RAW 264.7 cells were washed once with warm RPMI 1640 medium containing 10% fetal calf serum. TPCA-1 concentration was chosen according to Podolin’s report [16]. TPCA-1 and HA-AuNCs/T/P with the corresponding TPCA-1 concentration: 0.04 μg/mL, 0.1 μg/mL, and 0.2 μg/mL were added to the cells. RAW 264.7 cells were incubated for 30 min at 37 °C and then stimulated with 1.0 μg/mL lipopolysaccharide (LPS) for 24 h. Plates were centrifuged at 1000*g* for 10 min, and supernatants were removed and stored at 20 °C. The concentration of TNF-α and IL-6 were tested using an ELISA kit. The inhibition rate of cytokine was calculated according to a previous report [16], with some modifications:

Inhibition rate (%) = [(C_LPS-induced cytokine_ − C_Treatment_)/C_LPS-induced cytokine_] × 100%. (1)

### 2.7. Reactive Oxygen Species

ROS in RAW 264.7 cells were analyzed using the ROS assay kit, according to the manufacturer’s instructions and the previously reported method [17]. Briefly, RAW 264.7 cells (cell density 1.5 × 10^5^ cells/well) were seeded in 12 well plates and incubated for 24 h at 37 °C. AuNCs and HA-AuNCs/P (final concentration of AuNCs: 2.0 μg/mL) were added to the cells and then RAW 264.7 cells were stimulated with 1.0 μg/mL LPS for 24 h. Subsequently, 500 µL of cell culture medium (without FBS), containing 2′,7′-dichlorodihydrofluorescein diacetate (DCFH-DA) at a concentration of 10 µM, was added to the cells after they were washed three times with PBS. Cells were incubated for 20 min and washed several times with cell culture medium (without FBS) and PBS. Cells were collected and washed three times with PBS before ROS was determined using a flow cytometer (Accuri C6, BD Biosciences, San Diego, CA, USA).

### 2.8. Statistical Analysis

Data were expressed as mean ± standard deviation. All experiments were conducted three times. The differences in cytokine inhibition rates were analyzed via one-way analysis of variance (ANOVA) using SPSS (SPSS, version 21.0, IBM Inc., Amund, New York, USA). Differences were considered statistically significant when *P* < 0.05.

## 3. Results and Discussion

### 3.1. Synthesis and Characterization

**Figure 1:**
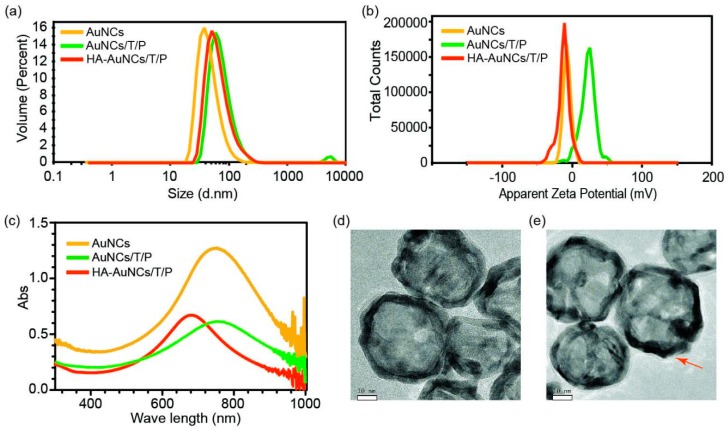
The characterization of AuNCs, AuNCs/T/P, and HA-AuNCs/T/P: (**a**) Size distribution and (**b**) zeta potential of AuNCs, AuNCs/T/P, and HA-AuNCs/T/P; (**c**) UV-Vis absorption spectra of AuNCs, AuNCs/T/P, and HA-AuNCs/T/P; and TEM micrograph of (**d**) AuNCs and (**e**) HA-AuNCs/T/P (scale bar: 10 nm).

The synthesized AuNCs, AuNCs/T/P, and HA-AuNCs/T/P were characterized for their size, zeta potential, UV spectra, and morphology. The z-average size and PDI of AuNCs, AuNCs/T/P, and HA-AuNCs/T/P were about 61 nm and 0.261 ± 0.007, 87 nm and 0.252 ± 0.003, and 82 nm and 0.232 ± 0.008, respectively (Figure 1a). The size of AuNCs increased after loading with TPCA-1 and modification with peptides. However, a size decrease was observed after HA conjugation. Zeta potential of AuNCs, AuNCs/T/P, and HA-AuNCs/T/P were −6.81 ± 0.29 mV, 22.00 ± 0.72 mV, and −14.1 ± 0.7 mV, respectively (Figure 1b). After modification with peptides and HA, the maximum value of absorption peak of AuNCs, AuNCs/T/P, and HA-AuNCs/T/P were 751, 755, and 678 nm, respectively (Figure 1c). Peptides are conjugated onto AuNC via the Au–S bond. Since the peptides were positively charged, modification with the peptides resulted in a change of zeta potential from negative to positive. In aqueous solution, peptides absorbed some water molecules and enlarged the diameter of AuNC. With HA modification, the zeta potential of AuNC changed back to negative because HA are negatively charged polysaccharides. HA modification also caused a decrease in particle size and a shift in UV absorption peak. One possible reason for this is that the negatively charged HA complexed with the oppositely charged AuNCs/T/P and condensed the peptide on the surface of AuNCs/T/P. This phenomenon is frequently observed in the condensation of DNA by cationic polyplex [18,19]. The condensation of AuNCs/T/P by HA form a compact layer on the surface of AuNCs that may disturb the UV absorption of AuNCs/T/P and cause the peak shift. TEM images showed the round morphology of AuNC and HA-AuNCs/T/P. Additional coating layers were also observed in HA-AuNCs/T/P, suggesting the formation of a compact layer on the surface of AuNCs (Figure 1d,e).

**Figure 2:**
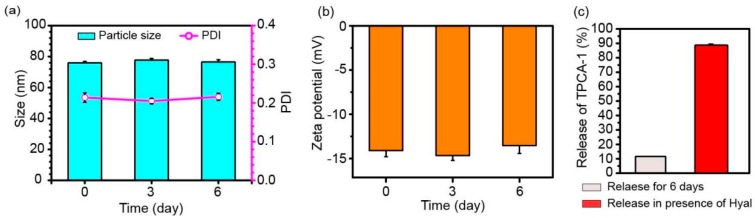
Stability and release of HA-AuNCs/T/P: (**a**) Particle size, PDI and (**b**) zeta potential of HA-AuNCs/T/P for 6 days at 4 °C; (**c**) Release of TPCA-1 from HA-AuNCs/T/P during six days’ storage or in the presence of Hyal. Data are presented as mean ± SD (*n* = 3).

The stability of HA-AuNCs/T/P was also explored by observing the size and zeta potential change of HA-AuNCs/T/P over six days. Particle size and the polydispersity of particle size were consistent during the six days (Figure 2a). There were also no significant changes of zeta potential values during the six days (Figure 2b). These data suggested a high storage stability in HA-AuNCs/T/P. Loading capacity and loading efficiency of TPCA-1 to AuNCs were determined as 0.145 μg/μg AuNCs and 26.1%, respectively. The amount of loaded TPCA-1 can meet the effective dosage for TPCA-1 application. During six days’ storage, little TPCA-1 was released, as shown in Figure 2c. However, in the presence of Hyal, TPCA-1 was released due to the degradation of HA on the surface of HA-AuNCs/T/P. These results indicate that the release of TPCA-1 may have been facilitated by the degradation of HA on the surface of HA-AuNCs/T/P under the action of intracellular Hyal, when HA-AuNCs/T/P were taken up into the cell.

### 3.2. In Vitro Study of HA-AuNCs/T/P

**Figure 3:**
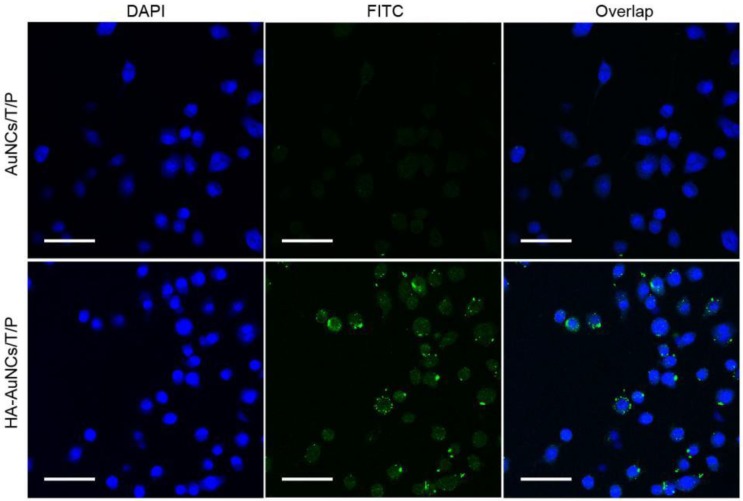
RAW 264.7 cells were incubated with AuNCs/T/P and HA-AuNCs/T/P for 4 h, and cellular uptake was recorded by laser confocal microscopy (scale bar: 25 μm).

The internalization of HA-AuNCs/T/P within inflamed cells is essential for their anti-inflammatory efficacy. Details of the cellular uptake were elucidated by confocal microscopy. The nuclei of RAW 264.7 cells were stained with DAPI and exhibited blue fluorescence. Peptides on AuNCs were labeled with FITC and emitted green fluorescence. Very little green fluorescence was observed in AuNCs/T/P-treated cells, whereas HA-AuNCs/T/P-treated cells showed strong green fluorescence. These results suggested that HA could facilitate the internalization of AuNCs/T/P into their targeted cells via receptor-mediated endocytosis (Figure 3). 

**Figure 4:**
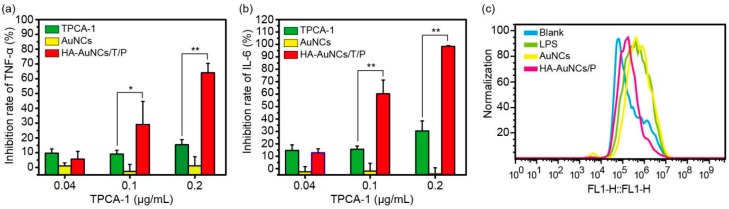
TPCA-1, AuNCs, and HA-AuNCs/T/P inhibit lipopolysaccharide (LPS)-induced (**a**) TNF-α and (**b**) IL-6 production; (**c**) ROS production in RAW 264.7 cells. Data are presented as mean ± SD (*n* = 3), * *p* < 0.05, ** *p* < 0.01.

TPCA-1 is an inhibitor for IKK in NF-κB signaling pathways [4]. Therefore, the application of TPCA-1 will downregulate NF-κB signaling pathways and reduce proinflammatory cytokines. As shown in Figure 4a,b, free TPCA-1 caused low inhibition of TNF-α and IL-6, at 0.1 and 0.2 μg/mL, whereas HA-AuNCs/T/P resulted in a significantly higher inhibition of TNF-α and IL-6 at 0.1 and 0.2 μg/mL of TPCA-1. The significant reduction of proinflammatory cytokines was mainly observed in prophylactic treatment. In prophylactic treatment, TPCA-1 blocks the phosphorylation of IKK in NF-κB signaling pathways and the downstream expression of proinflammatory cytokines. These results are consistent with the original report of TPCA-1 [16]. As seen in Figure 3, HA-AuNCs/T/P demonstrated a much higher intracellular uptake, suggesting that more TPCA-1 was delivered into the cells, which resulted in a higher anti-inflammation efficacy. The therapeutic treatment of TPCA-1 and HA-AuNCs/T/P was also explored. For the therapeutic treatment, macrophages were stimulated with LPS for 24 h to establish inflamed M1 macrophages and then treated with TPCA-1 and HA-AuNCs/T/P. However, no significant inhibition of TNF-α and IL-6 was shown (data not shown). A possible reason for this is that TPCA-1 only inhibits the production of more P50/P65 complex but cannot inhibit already-produced P50/P65 complexes from initiating the expression of cytokines. Podolin and co-workers reported effective inhibition in prophylactic treatment and therapeutic treatment in vivo [16]. TPCA-1 is effective, in vivo, in therapeutic treatments because TPCA-1 also participates in the inhibition of antigen-presenting neutrophils and the proliferation of T cells in vivo [16,20,21]. In future studies, the co-culture of macrophages with dendritic cells and T cells will be explored to further investigate the efficacy of therapeutic treatments involving TPCA-1. More studies will be needed to elucidate the treatment mechanisms of TPCA-1.

Previous studies have shown that ROS was one of the main contributors to the pathogenesis of RA and that gold nanoparticles can reduce the production of ROS [13,22]. To demonstrate the potential of AuNCs for RA therapy, ROS was also quantified in cells treated by AuNCs and HA-AuNCs/P. The results indicated that HA-AuNCs/P can reduce the production of ROS in inflammatory cells, when compared to AuNCs (Figure 4c). However, the quenching ROS efficacy of HA-AuNCs/P was undesirable. Mackey and co-workers observed that the presence of silver on the inner cavity of AuNCs can produce ROS by the oxidation of metallic silver in the cells [23]. This result may be the reason for the low activity of HA-AuNCs/P in quenching ROS. 

Although the results of this study suggest that HA-AuNCs/T/P could be utilized as potential efficient anti-inflammatory agents, in the future, we will need to investigate, in depth, the mechanisms of anti-inflammatory activity and in vivo anti-RA efficacy.

## 4. Conclusions

Due to the many advantages of AuNCs, they have been used for the treatment of inflammatory diseases like RA. In this study, AuNCs were used to carry the anti-inflammatory drug TPCA-1. The drug-loaded AuNCs were then modified with peptides and HA. The obtained HA-AuNCs/T/P were stable formulations that showed efficient intracellular uptake and good efficacy in the suppression of proinflammatory cytokines TNF-α and IL-6. Therefore, we conclude that HA-AuNCs/T/P are potential agents for anti-inflammatory drugs and that further investigation is warranted.
